# Microstructure and Mechanical Performance of Additively Manufactured Aluminum 2024-T3/Acrylonitrile Butadiene Styrene Hybrid Joints Using an AddJoining Technique

**DOI:** 10.3390/ma12060864

**Published:** 2019-03-14

**Authors:** Rielson Falck, Jorge F. dos Santos, Sergio T. Amancio-Filho

**Affiliations:** 1 Helmholtz-Zentrum Geesthacht, Center for Materials and Coastal Research, Institute of Materials Research, Materials Mechanics, Solid State Joining Processes, 21502 Geesthacht, Germany; rielson.falck@hzg.de (R.F.); jorge.dos.santos@hzg.de (J.F.d.S.); 2 Institute of Materials Science, Joining and Forming, BMVIT Endowed Professorship for Aviation, Graz University of Technology—TU Graz, Kopernikusgasse 24/1, Graz 8010, Austria

**Keywords:** AddJoining, FDM, additive manufacturing, aluminum 2024-T3, ABS, metal–polymer

## Abstract

AddJoining is an emerging technique that combines the principles of the joining method and additive manufacturing. This technology is an alternative method to produce metal–polymer (composite) structures. Its viability was demonstrated for the material combination composed of aluminum 2024-T3 and acrylonitrile butadiene styrene to form hybrid joints. The influence of the isolated process parameters was performed using the one-factor-at-a-time approach, and analyses of variance were used for statistical analysis. The mechanical performance of single-lap joints varied from 910 ± 59 N to 1686 ± 39 N. The mechanical performance thus obtained with the optimized joining parameters was 1686 ± 39 N, which failed by the net-tension failure mode with a failure pattern along the 45° bonding line. The microstructure of the joints and the fracture morphology of the specimens were studied using optical microscopy and scanning electron microscopy. From the microstructure point of view, proper mechanical interlocking was achieved between the coated metal substrate and 3D-printed polymer. This investigation can be used as a base for further improvements on the mechanical performance of AddJoining hybrid-layered applications.

## 1. Introduction

The substitution of conventional metals with lightweight materials is inevitable. Weight reduction and reliable mechanical performance are the driving forces in core industries, i.e., automotive and aerospace, to search for the next generation of materials and innovative production technologies.

The employment of multi-materials in a structure, where material, design, and manufacturing technique are the essential characteristics of the development of any engineering structural application, is a challenge. Joining technologies and additive manufacturing techniques complement the successful integration of material, design, and manufacturing technique [1]. Traditional joining methods, such as adhesive bonding and mechanical fastening, face technological limitations when joining dissimilar materials, such as metals and composites. For instance, the relatively long curing time of adhesive is a significant drawback in adhesive bonding. In recent years, new joining techniques have been investigated to develop hybrid joints to overcome the limitations of traditional joining methods. These include friction riveting [2], friction spot joining [3,4], injection clinching joining [5,6], ultrasonic joining [7,8], ultrasonic welding [9,10], and induction-heated joining [11].

An increased interest in the recent advances in the field of additive manufacturing (AM) is the flexibility to produce complex geometric parts, which are net-shaped with mechanical functionalities, such as in sandwich structures with AM honeycomb cores [12,13]. Combining the principles of joining and the polymeric AM, Falck et al. [14] recently introduced the AddJoining technology as an alternative method to produce metal–polymer (composite) layered structures. This technique is a patent application developed at Helmholtz-Zentrum Geesthacht, Germany (DE 102016121267A1). The process is inspired by AM and joining technology principles, where the new technique uses polymer 3D printing, e.g., fused deposition modeling (FDM), to add layers of polymer or composite on a metal substrate. Some of the advantages of AddJoining are geometric flexibility, such as honeycomb cores, a wide range of material combinations, and the production of complex parts. In the early phase of this technology, it is necessary to understand the adhesion forces and interactions between the metal and the polymer. In a previous publication [14], the authors briefly discussed the main bonding mechanisms that occur during the process, which are adhesion forces and mechanical interlocking at the metal–polymer interface.

The current work aims to evaluate the influence of different process parameters on the mechanical behavior of hybrid joints made by AddJoining. This exploratory study facilitates the first insights into the understanding and improvement of hybrid joints using AM means. Therefore, two well-established materials were selected for this study: aluminum alloy 2024-T3 and acrylonitrile butadiene styrene (ABS). The geometry selected to perform mechanical testing (lap shear test) was single-lap joint configuration. The one-factor-at-a-time (OFAT) approach and analysis of variance (ANOVA) were used to evaluate the significance of the selected parameters on the mechanical strength of the joints. Moreover, the microstructure of the joints and the fracture morphology of the specimens after failure were studied using optical microscopy (OM), laser confocal scanning microscope (LCSM), microcomputed tomography (µCT), and scanning electron microscopy (SEM).

### AddJoining Principles

**Figure 1:**
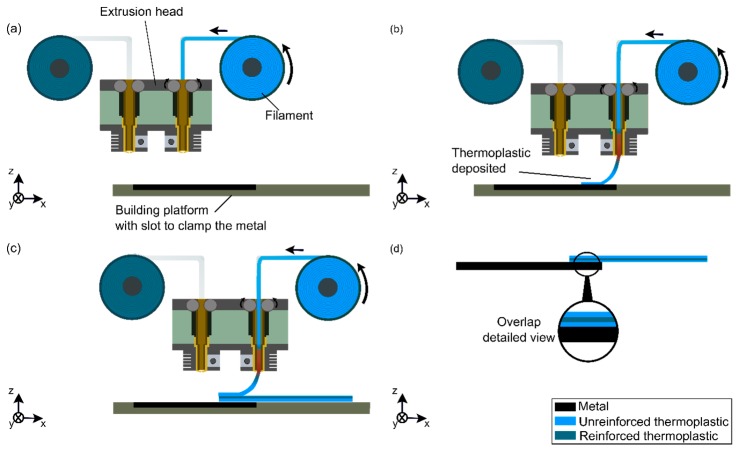
Schematic representation of the AddJoining process for layered metal–polymer composite hybrid structures: (**a**) initial setup, (**b**) deposition of the first polymer layer on the metal substrate, (**c**) deposition of the subsequent polymer layers, (**d**) final layered metal-polymer hybrid structure. Reproduced from Reference [14].

In the AddJoining method, fused deposition modeling (FDM) is used to form the polymeric part, where parts can be produced with complex geometries by depositing extruded material layer by layer on a substrate. This manufacturing technique is divided into three mains steps (Figure 1) [14]. Prior to starting to produce the metal–polymer hybrid part, AddJoining begins by slicing 3D CAD (Computer-Aided Design) data into layers. The first phase starts by fixing the metallic substrate on the build platform (Figure 1a). Subsequently, the polymer material is uncoiled slowly and guided to the extrusion head, where the resistive heated part is located closer to the nozzle. This is heated to high temperatures (above glass transition temperature or melting temperature) to decrease material viscosity. At this stage, the softened or molten material flows smoothly through the extrusion nozzle where the polymer material is deposited to form a full layer on top of the metal substrate (Figure 1b). Following each sequence, the building platform is lowered down by the thickness of one layer (in the Z-direction), while the extrusion head moves in a horizontal plane (the X-Y plane). The process is repeated, adding polymer layers on top of the previously consolidated polymer, until the final thickness of the polymeric part is achieved (Figure 1c). Finally, the metal–polymer layered joint is removed from the building platform (Figure 1d). Additional post-printing steps (e.g., thermomechanical treatment), such as hot isostatic pressing, may be applied to eliminate intrinsic voids in the layered component. This technique is usually used for metals and ceramics, but has been used as a possible technique to produce a homogeneous and defect-free material for 3D-printed polymers as well [15]. Note that post-processing was not considered in this work.

**Figure 2:**
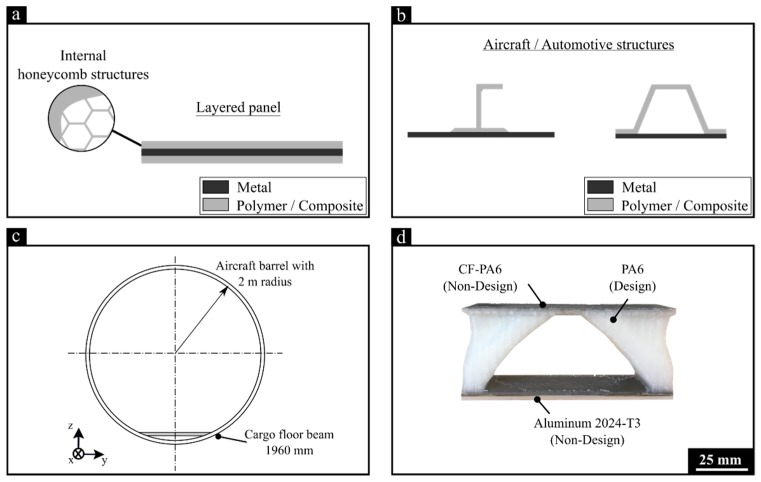
Example of potential AddJoining applications in transportation hybrid structures: (**a**) layered panel with internal honeycomb cores, (**b**) skin-stringer or B pillars for aircraft and automotive structures. Also, (**c**) schematic view of the fuselage barrel of a mid-size aircraft with (**d**) a topology-optimized 3D-printed cargo floor beam (scale 1:20).

The AddJoining has five controllable parameters, with four of these depending on the 3D printer selected. In this study, there are five controllable parameters: printing temperature (PT), road thickness (RT), deposition speed (DS), and number of contours (NC). The fifth controllable parameter is the ABS coating concentration (AC), where a homogeneous coating layer using the respective unreinforced filament materials is deposited on the metallic surface to promote better adhesion between the metal and subsequent printed polymer layers. Although only single-lap joints were evaluated in this study, AddJoining was conceived to produce rather complex 3D hybrid parts with integrated functionalities or optimized topologies. Figure 2a,b presents schematics of potential AddJoining parts, and Figure 2d shows an net-shape demonstrator part, which can be used in future topology and strength-to-weight optimized aircraft under floor beams (Figure 2c).

## 2. Materials and Methods

### 2.1. Aluminum 2024-T3

The aluminum alloy 2024-T3 used in this work was a rolled sheet with a thickness of 2 mm from Constellium, Issoire, France [16]. The metal alloy was selected thanks to its excellent mechanical properties and applicability in the transportation industry. Such material belongs to the 2xxx series of precipitation hardening aluminum alloy, where copper and magnesium are the major alloying elements. The physical and mechanical properties of the aluminum 2024-T3 used in this study are shown in Table 1.

### 2.2. Acrylonitrile Butadiene Styrene (ABS)

An unreinforced thermoplastic was selected and supplied by VShaper, Jasionka, Poland, in a spool with a filament 1.75 mm in diameter. The amorphous thermoplastic was ABS, which is a terpolymer with glass transition temperature of 105 °C [18,19,20]. Table 2 highlights some of the physical and mechanical properties of ABS. This polymer is a potential material for engineering applications to fulfill impact resistance, strength, and stiffness [21,22].

### 2.3. Surface Preparation

A pre-processing was necessary before AddJoining the parts, where the aluminum surface was treated to increase the roughness for better adhesion between the joining parts. The aluminum alloy was sandblasted with corundum (Al_2_O_3_), with the particle size ranging from 100 to 150 µm, with a pressure of six bar. The metal was located at an angle of 45° from the blasting nozzle and with a work distance of 200 mm. The surface pre-treatment was applied in the overlap area (12.5 mm × 25.5 mm) within 10 s. The pretreated samples were cleaned using pressurized air and immersed in ethanol for three minutes in an ultrasonic bath. The peak-to-valley distance surface roughness (Rz) were measured using LCSM. The average surface roughness of the aluminum part prior to sandblasting was 7 ± 0.8 µm. After this surface pre-treatment, the average Rz of the aluminum part 89 ± 0.3 µm increased up to 91% in comparison to the as received specimen surface. In other words, the sandblasting pre-treatment increased the surface roughness creating crevices, which support the formation of micro-mechanical interlocking between the deposited ABS coating layer and the Al 2024 surface.

After sandblasting, a coating was applied to the aluminum surface to increase the adhesion between the printed ABS and the sandblasted aluminum surface during the AddJoining process. Three different concentrations of ABS filament were evaluated, namely 5 wt.%, 15 wt.%, and 25 wt.%. To perform the coating, first ABS filament was dissolved in pure acetone at room temperature for 24 h. The solution was applied with a customized tool to spread it manually on the aluminum surface. The samples were subsequently dried in the horizontal position for five minutes at room temperature. The nominal coating thickness was measured after the AddJoining process using OM.

In this work, two phenomena occurred during the process. First, the applied coating formed a mechanical interlocking within the metallic sandblasted surface, where the polymer flowed and filled in the cavities. Second, the deposited polymer layer formed a bond with the coating layer [14].

### 2.4. Manufacturing Procedure

**Figure 3:**
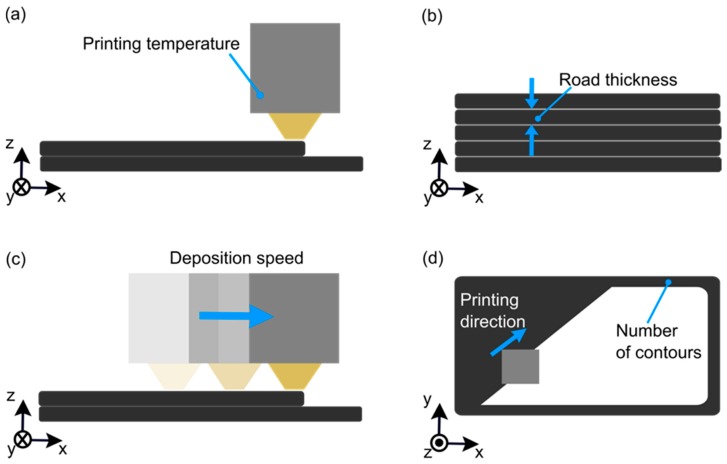
Representation of the AddJoining process parameters based on FDM: (**a**) printing temperature, (**b**) road thickness, (**c**) deposition speed, and (**d**) number of contours.

In this exploratory study, printing temperature, road thickness, deposition speed, ABS coating concentration, and the number of contours were selected as process parameters to produce aluminum 2024-T3/ABS hybrid joints using VShaper PRO (VShaper, Jasionka, Poland). Two parameters were constant along the OFAT, such as road angle in [−45°,45°] and building surface temperature at 115 °C. The first means the road deposition directions alternated for different layers between −45° and 45°. The five controllable process parameters are described as follows:

1. Printing temperature (PT) refers to the working temperature at the extruder head (Figure 3a), which was above the glass transition temperature or melting temperature, respectively, for amorphous and semi-crystalline thermoplastics.
2. Road thickness (RT) is the thickness of the consolidated road, which is the vertical distance between each layer (Figure 3b).
3. Deposition speed (DS) means the speed of the extruder head during operation (Figure 3c).
4. ABS coating concentration (AC) is the polymer concentration applied as the coating on the metallic surface.
5. The number of contours (NC) refers to the enclosed loops of road deposition in the filled-perimeter region (Figure 3d).

Table 3 shows the ranges for each process parameter. There were 11 combinations of process parameters for the OFAT approach. A total number of 33 aluminum 2024-T3/ABS hybrid joints were manufactured to investigate the ultimate lap shear force (ULSF). For each condition, three replicates were made, and the ULSF were averaged. With the similar process parameters, the mechanical performance of the aluminum 2024-T3/ABS hybrid joints was compared with the stand-alone printed ABS as the base material (BM) using FDM (hereafter, referred to ABS BM FDM).

### 2.5. Mechanical Performance

**Figure 4:**
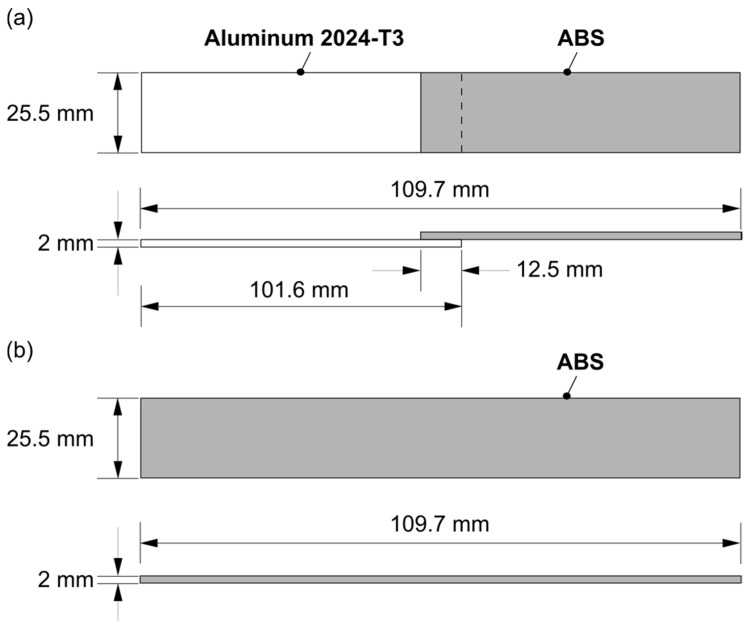
Scheme from the specimen geometry for the (**a**) AddJoining hybrid joints and (**b**) ABS BM FDM.

The joints were evaluated under quasi-static loading to assess the mechanical performance. Based on ASTM D3163-01, the single-lap shear test was performed in the universal testing machine Zwick/Roell 1478 (Zwick Roell, Ulm, Germany) at room temperature with a transverse speed of 1.27 mm/min. The AddJoining hybrid joint configuration had the specimen geometry of 101.6 mm × 25.5 mm × 2 mm with an overlap area of 12.5 mm × 25.5 mm (Figure 4a). For the comparison with the AddJoining hybrid joint, (Figure 4b), ABS BM FDM was produced, based on the total length (109.7 mm) from the single-lap joint configuration.

### 2.6. Microstructural and Fracture Surface Analysis

OM (DM IR microscope, Leica, Wetzlar, Germany) was used to analyze the microstructure of the joints. ImageJ, an open source software (Version 1.8, Public Domain), used for the images generated from the OM to evaluate the sizes of the pores in the printed polymer generated during the process.

Fracture morphology and surface coating formed on the metallic substrate were investigated using SEM (Quanta FEG 650, FEI, Hillsboro, OR, USA) for the optimum parameter combination with maximum ULSF. To eliminate charging effects beforehand, a vacuum sputter (Q150R ES, Quorum, Lewes, UK) was used to coat the sample with a layer of gold alloy before analysis. The sample was exposed for 30 s to a current of 65 mA.

### 2.7. Statistical Analysis of Mechanical Performance

The influence of the process parameters was evaluated using the one-factor-at-a-time (OFAT) approach. This approach was used to identify the range of process parameters in the early stage of a new process. It was conducted to evaluate the isolated effects of the process parameters. The parameters used to assess their influence on the selected response (ULSF) along with their corresponding levels are listed in Table 3. The effect of the process parameters on the response was performed using analysis of variance (ANOVA). Abibe et al. [6] used OFAT and ANOVA to investigate the influence of the injection clinching process parameters on the mechanical performance of Aluminum 2024-T351 and polyamide 6,6 reinforced with 30% short glass fibers.

A one-way ANOVA was conducted via use of the F-test, carried out on Minitab 14 software (Minitab Inc., State College, PA, USA). This method allows the rejection of the null hypothesis for the factors with no statistical significance on the response. Using this technique, the *p*-value < 0.05 (confidence level of 95%) was chosen as an indication of the significance of the parameter and the *f*-value was used to evaluate the effect of the process parameters.

### 2.8. Non-Destructive Testing

Microcomputed tomography (µCT) is a 3D computed tomography technique used to investigate damage and porosity analysis for additive manufacturing parts [23]. In this work, the internal structural information, e.g., pore size and pore distribution, were evaluated using YXLON Cougar EVO (Yxlon, Hamburg, Germany), which is a microcomputed tomography method for seven conditions (condition one to condition seven from Table 4) only for the ABS BM FDM. There are two reasons for this initial approach. First, the presence of a metallic part in the hybrid joint led to insufficient resolution and mistaken identification within the polymeric volume. Further investigation needs to be performed to identify the ideal radiation source. For this initial evaluation, only the ABS BM FDM was considered to understand the influence of the process parameter on the internal structural formation. The outcome of this investigation can aid our understanding of the impact of the process parameter on the mechanical performance of AddJoining hybrid joints. The scanned volume considered was nearly 125,000 mm^3^; however, the physical volume size was 25 mm × 30 mm × 2 mm, excluding the air surrounding the sample. The specimen was fixed on a rotational stage, and the distance between the rotation axis and the radiation source was set to approximately 10 mm. The X-ray tube voltage was set to 40 kV, and X-ray tube current was set to 40 µA for all the specimens. An image was acquired per degree during X-ray projection images, and the 3D image was reconstructed using the image processing unit of the X-ray CT system. VGStudio Max 3.1 (Volume Graphics, Heidelberg, Germany) was used and a Gaussian filter was applied to remove the noise from the scanning method.

## 3. Results

### 3.1. Influence of the AddJoining Process Parameters on the ULSF

Single-lap joints are typically affected by the eccentricity on the load path. Eccentric load means a nonsymmetrical concerning the central axis, thereby producing a bending moment during the loading. Thus, a single lap joint in tension leads to large deflections, and the relationship between the bending moment in cross-section and the applied tensile force is nonlinear. With an increase in the tensile loading, stress analysis of the single lap joint becomes highly nonlinear. Hence, the state of the stress in the aluminum 2024-T3/ABS hybrid joints is not purely axial stress but shear and peel stresses also appear. In contrast, for ABS BM FDM, the axial stress is uniform as a result of the load application direction. Therefore, it is expected that ABS BM FDM leads to higher ductility than aluminum 2024-T3/ABS hybrid joints. ULSF is the primary response in the OFAT. However, to help with the discussions, the displacement at break (DaB) for each condition was also taken into account to compare the ductility between the aluminum 2024-T3/ABS hybrid joints (Table 5) and the ABS BM FDM (Table 4). Nevertheless, the ULSF values were added to the strength for condition eight and condition nine, based on ABS BM FDM from condition seven because of the similarity in the process parameters (Table 4). Note that in this case, no direct comparison was made between the strength of the joint with the base material because no solution was necessary to produce ABS BM using FDM.

The ANOVA technique was used to assess the effects of the process parameters. Based on the OFAT design, the parameters studied were evaluated independently for aluminum 2024-T3/ABS hybrid joints (Table 6) and ABS BM FDM (Table 7). For the mechanical performance, all parameters except PT were statistically significant within the confidence level, where the probability value (*p*-value) was below 0.05. According to the F-test in ANOVA, the order of significance is different for single-lap joint and ABS BM FDM. For aluminum 2024-T3/ABS hybrid joints, the order of relevance is based on the *f*-value, where the highest significant parameter was DS, followed by AC, NC, and RT having the lowest significance. The order was slightly different for ABS BM FDM, where NC, DS, and RT were, respectively, had the highest to the lowest order of relevance. In the following section, the influence of each process parameter on the ULSF is discussed separately.

#### 3.1.1. Printing Temperature

**Figure 5:**
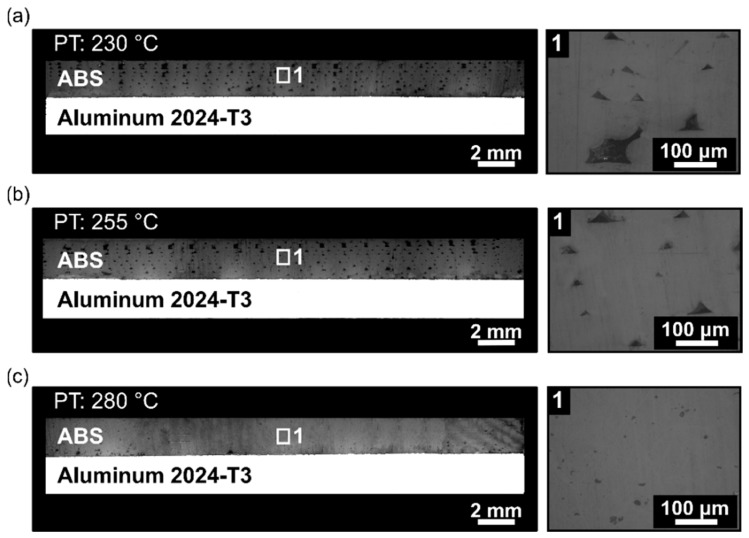
Microstructure of the aluminum 2024-T3/ABS hybrid joints manufactured with different printing temperatures, with detailed view of the pores formed within the ABS part for (**a**) condition one, PT: 230 °C, (**b**) condition two, PT: 255 °C, and (**c**) condition three, PT: 280 °C (the following parameters RT: 0.2 mm, DS: 40 mm/s, AC: 15 wt.%, and NC: 2 were fixed for the conditions investigated).

Figure 5 shows the cross-section of the joint for each printing temperature that was investigated. A detailed view of the polymeric part was considered to highlight the presence of pores. The pore surface area of the polymeric cross-sectional view was investigated to evaluate the average pore size. From the images, increasing the printing temperature from 230 °C (Figure 5a) to 280 °C (Figure 5c) led to a decrease of 320% in the void area from 1314 ± 81 µm^2^ (Figure 5a (1)) to 314 ± 27 µm^2^ (Figure 5c (1)). In the additive manufacturing of amorphous thermoplastics such as ABS, the primary bonding mechanisms between the printed layers are by thermal fusion and interlayer bonding. Mendelson [24] correlated the melt viscosity dependence of ABS with temperature. The author reported that a variation in temperature reduced the melt viscosity up to 50% (4600 Pa·s (230 °C) and 2500 Pa·s (260 °C)), where the bonding between the layers is thermally driven by the polymer viscous flow process [25]. Keeping the temperature above the glass transition temperature assured us that there would be good bonding between successively deposited layers.

**Figure 6:**
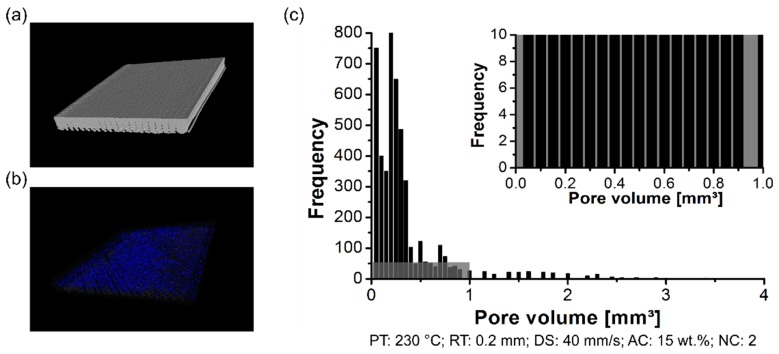
Pore distribution on the 3D-printed ABS for condition one (PT: 230 °C, RT: 0.2 mm, DS: 40 mm/s, AC: 15 wt.%, and NC: 2): (**a**) scanned volume, (**b**) pores distribution, and (**c**) pore volume histogram.

**Figure 7:**
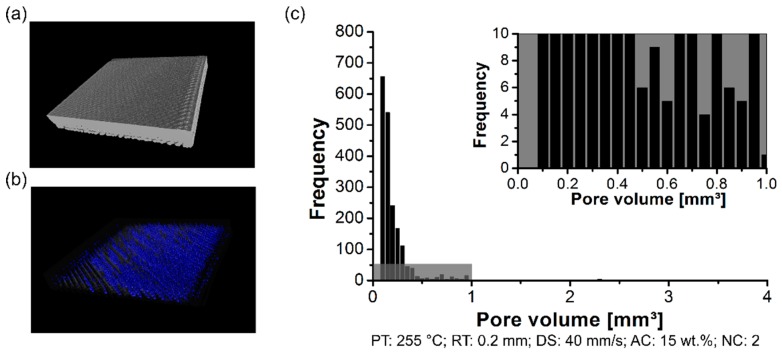
Pore distribution on the 3D-printed ABS for condition one (PT: 255 °C, RT: 0.2 mm, DS: 40 mm/s, AC: 15 wt.%, and NC: 2): (**a**) scanned volume, (**b**) pores distribution, and (**c**) pore volume histogram.

**Figure 8:**
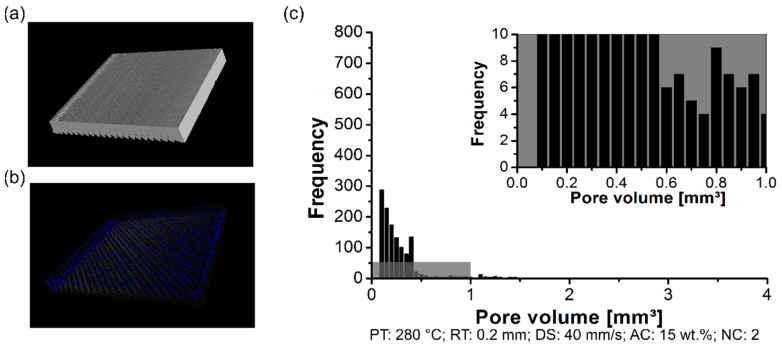
Pore distribution on the 3D-printed ABS for condition one (PT: 280 °C, RT: 0.2 mm, DS: 40 mm/s, AC: 15 wt.%, and NC: 2): (**a**) scanned volume, (**b**) pores distribution, and (**c**) pore volume histogram.

The formation of the pores was analyzed using a non-destructive test to determine the pores content within the 3D-printed parts, their distribution, and size. By changing the PT, the distribution of the pore volume of the polymeric part was affected. The formation of the pores was investigated in the scanned volume for condition one (PT: 230 °C, Figure 6a), condition two (PT: 255 °C, Figure 7a), and condition three (PT: 280 °C, Figure 8a). Each graph contains a highlighted section against a gray background, where it corresponds to a highlighted graph with pore frequencies (from 0 to 10) and pore volume (from 0 mm^3^ to 1 mm^3^). This scale range was selected to conduct a qualitative comparison across all conditions investigated in this work.

For the three conditions mentioned here, it reveals pores within the scanned 3D-printed polymeric part (Figure 6b, Figure 7b and Figure 8b). Notably, the amount of average pore volume size decreased by nearly 40% by increasing the PT from 230 °C (0.05 mm^3^ to 2.5 mm^3^) to 255 °C (0.05 mm^3^ to 1.25 mm^3^). Moreover, the pore volume size did not experience abrupt changes by increasing the PT from 255 °C to 280 °C, i.e., the voids kept constant in the range of 0.05 mm^3^ to 1.25 mm^3^. The pores were generally smaller and less frequent by increasing the PT from 230 °C (Figure 6c) to 280 °C (Figure 8c). It reflects the fact that part density, which is the ratio between the total pore volume and total material volume for each condition, is influenced by changing the PT. Preliminary results showed a decrease in the pore content from 4 ± 1% (at 230 °C) to 1 ± 0.4% (at 280 °C).

**Figure 9:**
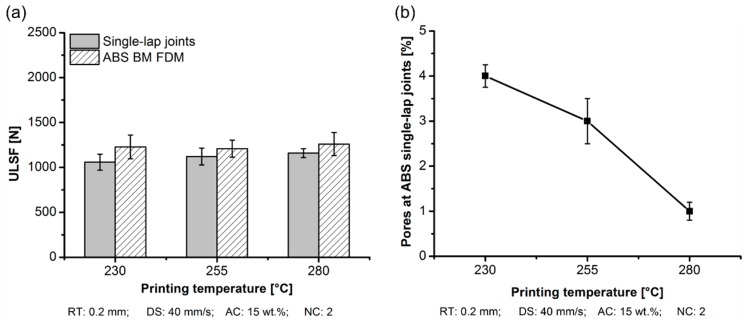
Influence of the printing temperature on the aluminum 2024-T3/ABS hybrid (single-lap joints) and ABS BM FDM (**a**) ULSF and (**b**) pores formation at the ABS part; (condition one, PT: 230 °C; condition two, PT: 255 °C; condition three, PT: 280 °C; the following parameters RT: 0.2 mm, DS: 40 mm/s, AC: 15 wt.%, and NC: 2 were fixed for the conditions investigated).

The strength of the AddJoining part was influenced by the presence of voids in the 3D-printed polymeric part. The aluminum 2024-T3/ABS hybrid joints demonstrated an increase of 9% on increasing the PT from 230 °C (ULSF: 1058 ± 89 N) to 280 °C (ULSF: 1159 ± 50 N), as shown in Figure 9a. An increase in the PT increased the mechanical performance of ABS BM FDM by 5%, where the condition with 230 °C (condition one) achieving 1228 ± 132 N and 280 °C (condition three) achieving 1259 ± 128 N. The difference in the ductility between aluminum 2024-T3/ABS hybrid joints and ABS BM FDM was previously explained. As shown in Table 5, the average DaB of the aluminum 2024-T3/ABS hybrid joints increased (approximately 8%) by changing the PT from 230 °C (DaB: 1.2 ± 0.3 mm) to 280 °C (DaB: 1.3 ± 0.2 mm). However, ABS BM FDM deformation increased by nearly 20%, respectively from 4.7 ± 0.7 mm to 5.7 ± 0.5 mm, and respectively from 230 °C to 280 °C (Table 4). The variations in ductility and strength were correlated with the reduction in the voids content in the 3D-printed polymeric part (Figure 9b). Preliminary results showed a decrease in the pore content from 4 ± 1% (at 230 °C) to 1 ± 0.4% (at 280 °C).

For polymer materials in the 3D-printed part, the increase in printing temperature allows chain diffusion and polymer entanglement across the interface formed between adjacent consolidated roads or layers, leading to a stronger bonding formation [26].

#### 3.1.2. Road Thickness

PT was fixed at 280 °C, as observed from the previous section. The following parameters—DS: 40 mm/s, AC: 15 wt.%, and NC: 2—were also fixed to continue with the investigation on the mechanical performance (ULSF). RT is a parameter used to investigate the influence on the mechanical performance by varying in 0.1 mm (condition four), 0.2 mm (condition three), and 0.3 mm (condition five). The bonding between the adjacent roads and layers influenced the strength of a 3D-printed part. As previously commented on PT, for polymer materials, a sintering process occurred at the interface during the road deposition. Hence, the bonding mechanism was primarily caused by adhesion between the roads and layers.

**Figure 10:**
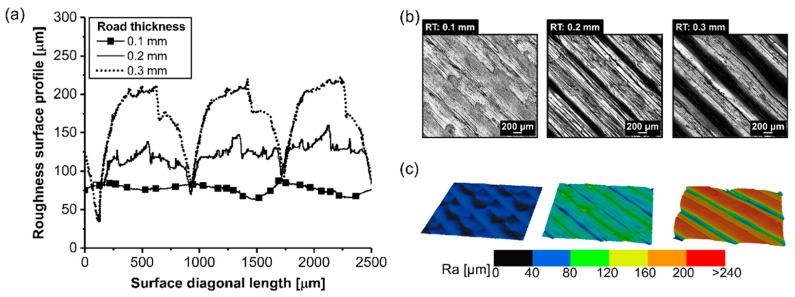
Influence of the surface roughness on the 3D-printed ABS for different road thicknesses (**a**) roughness surface profile, (**b**) surface image, and (**c**) 3D-image from the surface (condition four, RT: 0.1 mm; condition three, RT: 0.2 mm; condition five, RT: 0.3 mm; the following parameters PT: 280 °C, DS: 40 mm/s, AC: 15 wt.%, and NC: 2 were fixed for the conditions investigated).

The surface quality was evaluated to influence the RT in the surface roughness (Figure 10a). It is possible to identify a trend on the surface roughness, where in the thicker RT (0.3 mm), a lack of bonding typically existed, also known as air gaps (Figure 10b), with an average surface roughness of 240 ± 22 µm. In contrast, a thinner RT (0.1 mm) resulted in a smooth surface with nearly no gaps visible. By decreasing the RT from 0.3 mm to 0.1 mm, this decreased the average surface roughness by 67% (from 240 ± 22 µm to 80 ± 17 µm). This result is in qualitative agreement with the findings of Anitha et al. [27] where the authors found that decreasing the road thickness decreases the surface roughness and impacts the surface quality.

**Figure 11:**
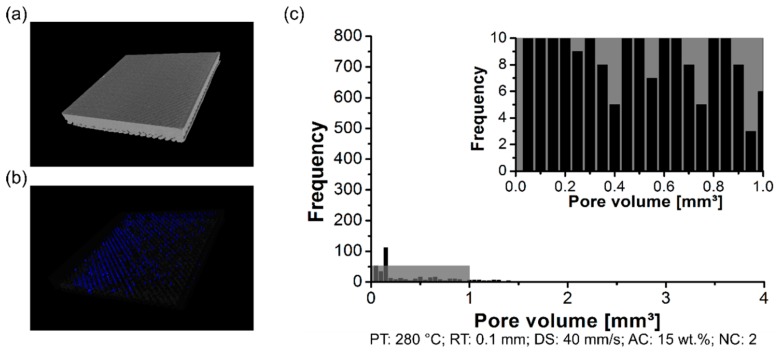
Pore distribution on the 3D-printed ABS for condition four (PT: 280 °C, RT: 0.1 mm, DS: 40 mm/s, AC: 15 wt.%, and NC: 2): (**a**) scanned volume, (**b**) pores distribution, and (**c**) pore volume histogram.

**Figure 12:**
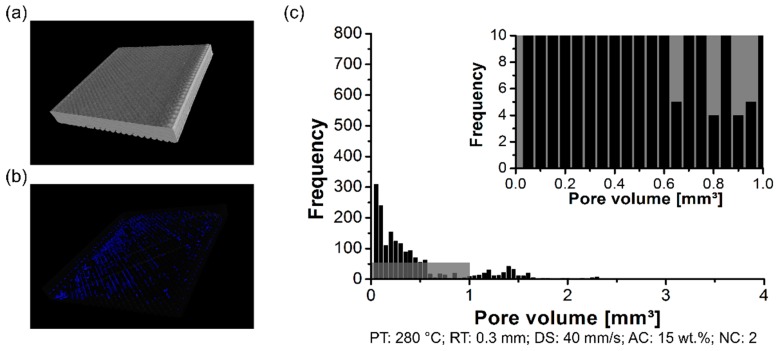
Pore distribution on the 3D-printed ABS for condition four (PT: 280 °C, RT: 0.3 mm, DS: 40 mm/s, AC: 15 wt.%, and NC: 2): (**a**) scanned volume, (**b**) pores distribution, and (**c**) pore volume histogram.

By measuring the pore volume in each RT condition, cited here, it was possible to correlate the pore content with the surface roughness. The scanned volume for condition three (RT: 0.2 mm) has been previously commented on in PT condition (Figure 8a). Moreover, condition four (RT: 0.1 mm) and condition five (RT: 0.3 mm) are respectively displayed in Figure 11a and Figure 12a. For the three conditions cited here, it revealed no concentrated area of pores, but instead a random distribution across the scanned volume for the formation of pores (RT: 0.1 mm, Figure 11b; RT: 0.2 mm, Figure 8b; RT: 0.3 mm, Figure 12b). The pore volume drastically decreased by 55% by decreasing the RT from 0.3 mm (0.05 mm^3^ to 2.25 mm^3^; Figure 12c) to 0.1 mm (0.05 mm^3^ to 1.25 mm^3^, Figure 11c). Moreover, it is relevant to comment that the frequency of pores was reduced by decreasing the RT. It reflects the point that part density—in this study, this is the ratio between the total pore volume and total material volume for each condition—was influenced by changing the RT. It indicated that the thinner RT (0.1 mm) was compactly stacked together, leading to a better interlayer bonding when compared to a thicker RT (0.3 mm).

**Figure 13:**
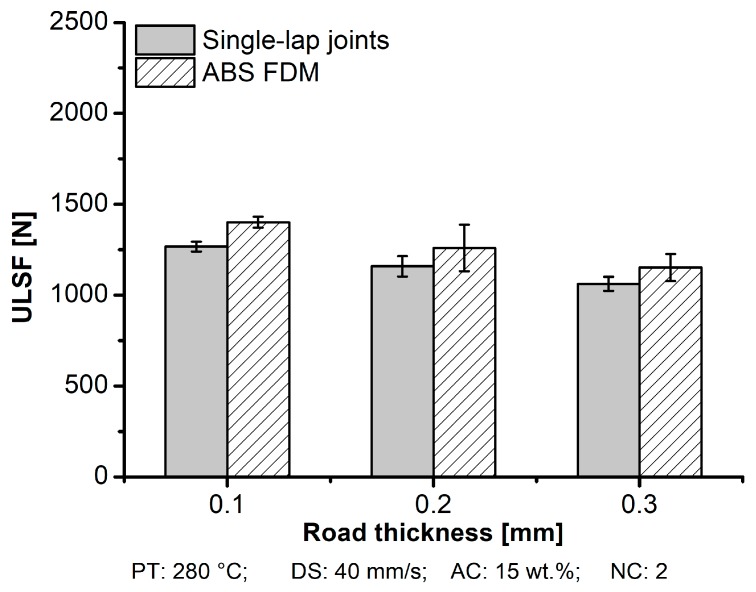
Influence of the road thickness on ULSF for aluminum 2024-T3/ABS hybrid joints (single-lap joints) and ABS BM FDM; (condition four, RT: 0.1 mm; condition three, RT: 0.2 mm; condition five, RT: 0.3 mm; the following parameters PT: 280 °C, DS: 40 mm/s, AC: 15 wt.%, and NC: 2 were fixed for the conditions investigated).

As discussed earlier, increasing the RT increased the surface roughness in the external part and pore accumulation in the internal structure. Hence, it could be expected that because of the presence of pores in between the layers, this could lead to stress concentration and a possible location to form cracks. Therefore, Figure 13 depicts the relationship between RT and the mechanical performance of aluminum 2024-T3/ABS hybrid joints and ABS BM FDM, which were highly influenced by the RT parameter. The difference in the mechanical performance between the single-lap joint and tensile specimen was explained in the first few sections. For aluminum 2024-T3/ABS hybrid joints, reducing the RT from 0.3 mm (ULSF: 1267 ± 27 N) to 0.1 mm (ULSF: 1062 ± 39 N) improved the mechanical performance by 19%. A similar trend was observed for the ABS BM FDM, where an improvement of 22% in the mechanical performance was achieved by changing the RT from 0.3 mm (ULSF: 1152 ± 75 N) to 0.1 mm (ULSF: 1401 ± 30 N). Table 4 shows that the average DaB of the aluminum 2024-T3/ABS hybrid joints decreased (approximately 15%) by changing the RT from 0.1 mm (DaB: 1.5 ± 0.5 mm) to 0.3 mm (DaB: 1.3 ± 0.3 mm). A similar reduction in ductility was observed for ABS BM FDM of nearly 14% (4.8 ± 0.3 mm to 4.2 ± 0.2 mm) from 0.1 mm to 0.3 mm, respectively.

3D-printed part integrity is primarily conceived through bonding with subsequent layers. Thus, the results show that smaller road thicknesses led to small pore formation, where the thickness of the polymer improved the interfacial bonding strength, with a lower layer thickness contributing to dissipate the stress in an easier manner. It, therefore, aids our understanding of the improvement in mechanical performance within the parameter conditions studied in this work. Sood et al. [28] concluded that an increase of around 15% in mechanical performance could be achieved when building with thinner road thickness. Wu et al. [29] and Shubahm et al. [30] observed that the presence of microvoids was smaller for thinner road thickness. Ning et al. [31] also observed a similar effect, where the lower thickness led to considerable inter-bonding strength.

#### 3.1.3. Deposition Speed

Considering the effect of PT and RT fixed at 280 °C and 0.1 mm, respectively, because of high mechanical performance and low presence of pores in the internal structure of the 3D-printed polymer, the following parameters—AC: 15 wt.% and NC: 2—were also fixed to continue with the investigation on the mechanical performance (ULSF). In this work, DS is a parameter used to investigate the influence on the mechanical performance by varying at 20 mm/s (condition six), 40 mm/s (condition four), and 60 mm/s (condition seven).

**Figure 14:**
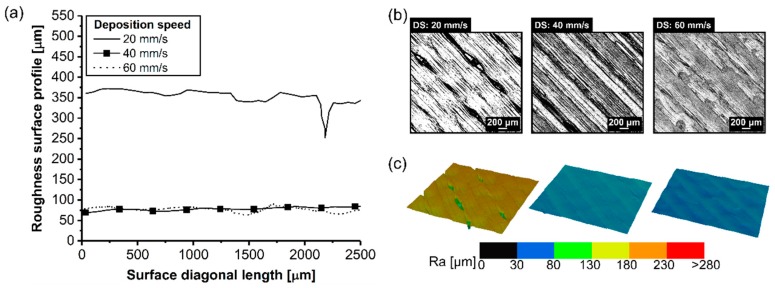
Influence of the surface roughness on the 3D-printed ABS for different deposition speeds (**a**) roughness surface profile, (**b**) surface image, and (**c**) 3D-image from the surface (condition six, DS: 20 mm/s; condition four, DS: 40 mm/s; condition seven, DS: 60 mm/s; the following parameters PT: 280 °C, RT: 0.1 mm, AC: 15 wt.%, and NC: 2 were fixed for the conditions investigated).

The surface quality was evaluated to understand the influence the DS had on the surface roughness (Figure 14a). Within the parameter range studied, a lower DS (20 mm/s) led to gaps between the roads (Figure 14b) with an average surface roughness of 302 ± 77 µm. In contrast, DS equal to and above 40 mm/s resulted in a smooth surface, with nearly no gap visible (DS: 40 mm/s; 80 ± 17 µm) and (DS: 60 mm/s; 70 ± 3 µm). Hence, by increasing the DS from 20 mm/s to 60 mm/s, the average surface roughness decreased four-fold. It is important to emphasize that the road was deposited into the coated metal substrate, neighboring consolidated road, and on top of the consolidated layer. Typically, the road could lose the heat via conduction to the consolidated neighboring road and the road below and by convection to the surrounding air in the envelope environment. Hence, the road was cooling, and the viscosity increased until a solid state was reached. However, the consolidated road temperature increased when a new road was deposited. The effect of DS associated with RT could increase adhesion forces because a thinner RT increased the interfacial bonding strength and stress flow was dissipated through the interface easier [32].

**Figure 15:**
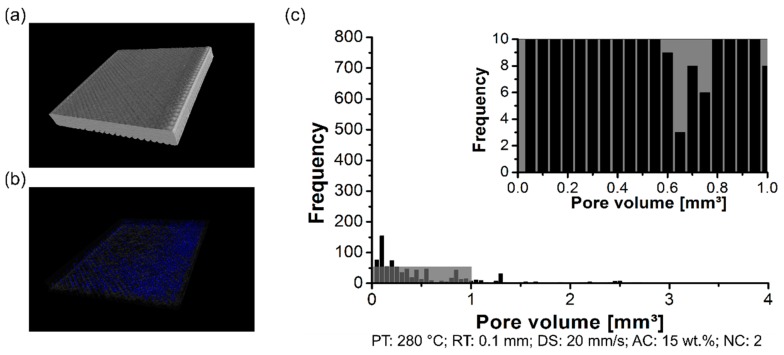
Pore distribution on the 3D-printed ABS for condition six (PT: 280 °C, RT: 0.1 mm, DS: 20 mm/s, AC: 15 wt.%, and NC: 2): (**a**) scanned volume, (**b**) pores distribution, and (**c**) pore volume histogram.

**Figure 16:**
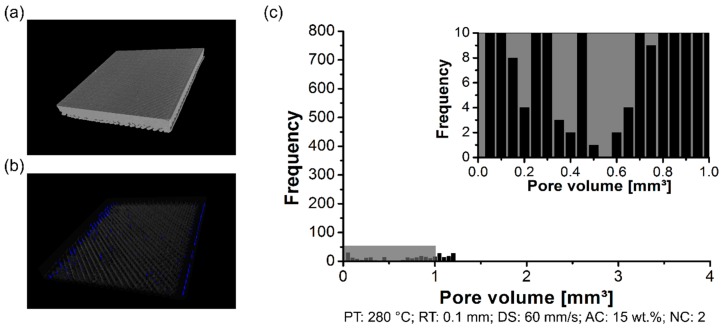
Pore distribution on the 3D-printed ABS for condition seven (PT: 280 °C, RT: 0.1 mm, DS: 60 mm/s, AC: 15 wt.%, and NC: 2): (**a**) scanned volume, (**b**) pores distribution, and (**c**) pore volume histogram.

**Figure 17:**
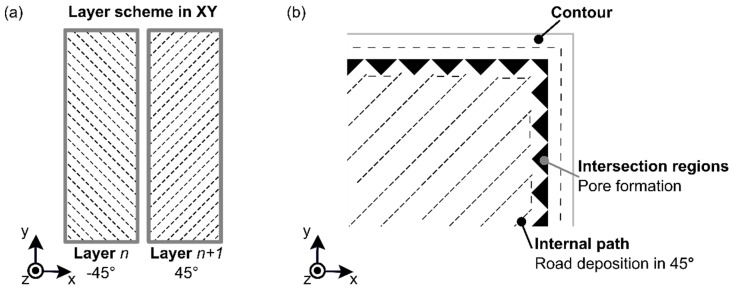
(**a**) Schematic view of typical 3D-printed layers with (**b**) a detail schematic on the pore formation in the intersection region between road deposition at 45° and contour.

As presented in previous sections, the measurements of the pore volume in each DS condition are cited here. The scanned volume for condition four (DS: 40 mm/s) has been previously commented on regarding the RT condition (Figure 11a). In this section, the following condition six (DS: 20 mm/s, Figure 15a) and condition seven (DS: 60 mm/s, Figure 16a) showed no presence of a preferable concentrated area of pores in the polymeric part (DS: 20 mm/s, Figure 15b; DS: 40 mm/s, Figure 11b). In contrast, the condition with a high DS at 60 mm/s showed a low concentration of pores in the volume edge of the 3D-printed polymer part (Figure 16b). Figure 17a shows the orientation in odd and even layers in the XY-plane to explain the concentration of pores in the edges. A detailed schematic view in Figure 17b shows the nozzle trajectory, where the infill road deposition at 45° created a region with a lack of material when it reached the outer contour. Eiliat and Urbanic [33] reported a similar observation, and, by applying an algorithm to change the road deposition trajectory, it was possible to minimize the voids in the preferable regions.

Furthermore, for all conditions cited in this section, the pore volume was kept constant in the range of 0.05 mm^3^ to 1.25 mm^3^. However, it is significant to note that the number of pores was reduced drastically for higher DS (condition seven, Figure 16c), with no pore frequency above 50. In the literature described [18,34], the temperature of the road decay on a time scale of two seconds for ABS was near to the glass transition temperature for the FDM process. Seppala et al. [35] observed that interlayer bonding is sensitive to the printing temperature and associated with the deposition speed; this can increase the interface bonding layer between polymer and polymer. As presented in this section, and considering the parameter range, it reveals that a higher DS does not give enough time to the deposited road for consolidation before depositing the following road. Hence, the previous road remains softened upon the deposition of the subsequent road. It facilitates the intermolecular diffusion in nearby roads. Therefore, it enables a better interlayer bonding in the interface polymer-to-polymer, consequently reducing the microvoids.

**Figure 18:**
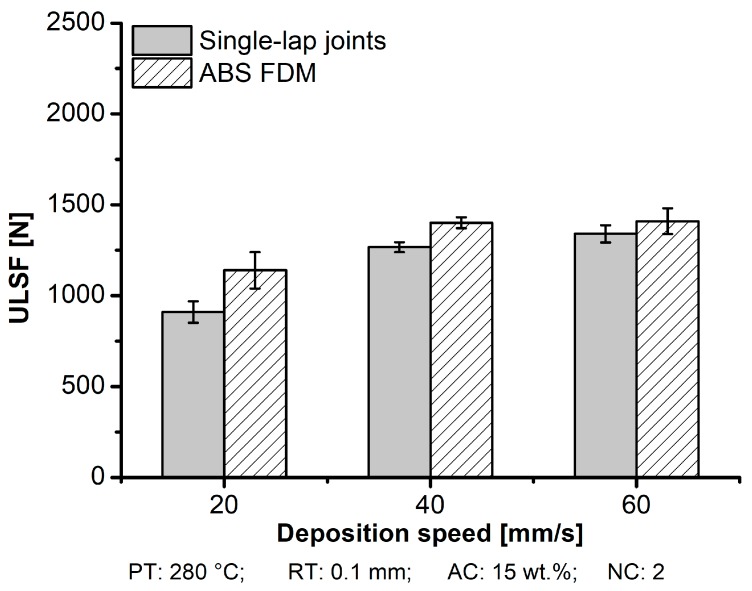
Influence of the deposition speed on ULSF for the aluminum 2024-T3/ABS hybrid joints (single-lap joints) and ABS BM FDM (condition four, RT: 0.1 mm; condition three, RT: 0.2 mm; condition five, RT: 0.3 mm; the following parameters PT: 280 °C, DS: 40 mm/s, AC: 15 wt.%, and NC: 2 were fixed for the conditions investigated).

It has been shown that DS reduces pore accumulation due to high interlayer bonding formation. It intrinsically plays a significant role in the mechanical performance (Figure 18). For aluminum 2024-T3/ABS hybrid joints and ABS BM FDM, increasing the DS led to high mechanical performance. For the aluminum 2024-T3/ABS hybrid joints, the ULSF increased 33% from 910 ± 59 N to 1340 ± 47 N when the DS was increased from 20 mm/s (condition six) to 60 mm/s (condition seven). For tensile specimens, an improvement of 24% was achieved by changing the DS from 20 mm/s (ULSF: 1142 ± 33 N) to 60 mm/s (ULSF: 1410 ± 71 N). As shown in Table 5, the average DaB of the aluminum 2024-T3/ABS hybrid joints decreased by 50% at lower DS (20 mm/s; DaB: 0.9 ± 0.1 mm) in comparison to higher DS (60 mm/s; DaB: 1.8 ± 0.3 mm). For the ABS BM FDM (Table 4), the ductility was also improved by 37%, changing in DS from 4.5 ± 0.6 mm to 6.2 ± 0.9 mm for 20 mm/s to 60 mm/s, respectively.

#### 3.1.4. ABS Coating Concentration

The fourth parameter, AC, was studied after fixing PT at 280 °C, RT at 0.1 mm, and DS at 60 mm/s, which improved the mechanical properties of the aluminum 2024-T3/ABS hybrid joints. AC varied as follows: 5 wt.% (condition eight), 15 wt.% (condition seven), and 25 wt.% (condition nine).

**Figure 19:**
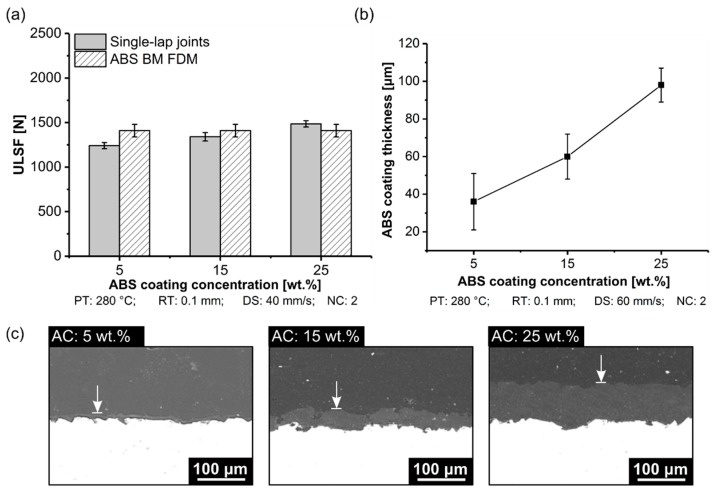
Influence of the ABS coating concentration on the aluminum 2024-T3/ABS hybrid (**a**) ULSF, (**b**) ABS coating thickness, and (**c**) detailed view from the ABS film formation (condition eight, AC: 5 wt.%; condition seven, AC: 15 wt.%; condition nine, AC: 25 wt.%; the following parameters PT: 280 °C, RT: 0.1 mm, DS: 60 mm/s, and NC: 2 were fixed for the conditions investigated).

The aluminum 2024-T3/ABS hybrid joint achieved the highest ULSF for condition nine (AC: 25 wt.% ABS) (Figure 19a). Based on the experiment, it resulted in an improvement of 30% in the mechanical aspect by increasing the AC from 5 wt.% (ULSF: 1142 ± 35 N) to 25 wt.% (ULSF: 1486 ± 36 N). The ULSF values were added to the strength for condition eight, and condition nine was added, based on ABS BM FDM from condition seven due to the similarity with the process parameters (Table 4). Please note that in this case, no direct comparison was made between the strength of the joint with the base material because no solution was necessary to produce ABS BM using FDM. Table 5 shows that the ductility was compromised with a low concentration of ABS (5 wt.%), where a premature failure of the joint occurred (DaB: 0.8 ± 0.4 mm). Contrariwise, a high concentration of ABS in the coating allowed for a high deformation in the joint (DaB: 1.9 ± 0.2 mm).

The thickness of the ABS coating on the metallic surface was measured. In this experiment, the thinnest AC (5 wt.% ABS) reached a coating thickness of 36 ± 15 µm, approximately 63% lower than the thickest AC (25 wt.% ABS), which achieved 98 ± 9 µm (25 wt.% ABS) (Figure 19b). The mechanical performance decreased for coating thicknesses below 100 µm. This thickness dependency is not exclusive for AddJoining principles, where the results of this work give a hint that the AC coating layer behaved similarly to adhesive bonding in the single-lap joint. Typically, the stress distribution acting in the interface is associated with axial and bending stress. Hence, the thinner coating layer (5 wt.% ABS) led to higher stresses because of the lower mass interaction in the interface. On the contrary, a thicker coating layer (25 wt.% ABS) had more mass to deform in the interface metal-polymer, leading to plastic deformation; this may have resulted in low shear and peel stresses.

**Figure 20:**
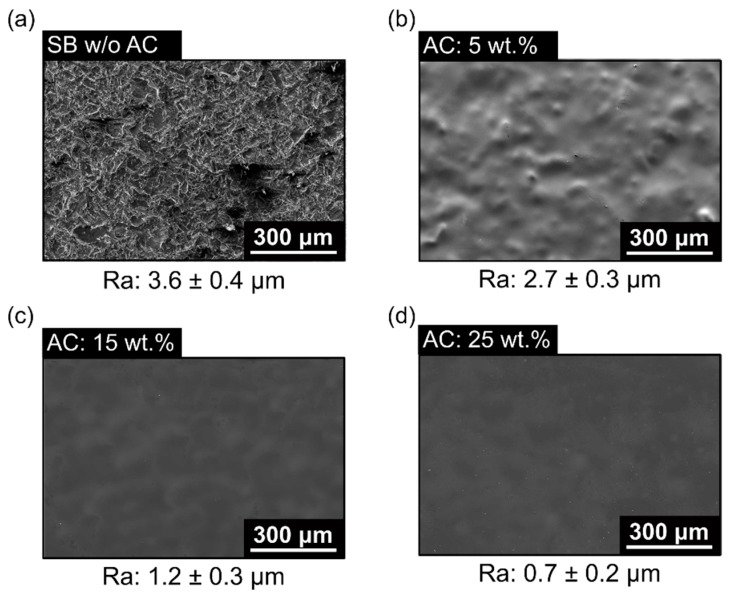
Surface roughness on the sandblasted aluminum surface: (**a**) without coating, (**b**) with 5 wt.% (condition eight), (**c**) with 15 wt.% (condition seven), and (**d**) with 25 wt.% (condition nine); (the following parameters PT: 280 °C, RT: 0.1 mm, DS: 60 mm/s, and NC: 2 were fixed for the conditions investigated).

The effect of the ABS coating thickness can be considered similar to an adhesive thickness on the mechanical performance of single-lap joint (Figure 19c). Objois et al. [36] observed that a lower adhesive thickness below 50 µm led to sudden decreases in mechanical performance. The authors reported that local stress concentration caused by the non-uniform stress distribution in the adhesive layer led to the premature adhesive failure of the joint. Hence, the stress levels increase with decreasing adhesive thickness. In fact, the thinner coating layer probably causes local stress concentration, because of the non-uniformity in the coating layer, which resulted in the roughness of the metallic part (Figure 20b) with 75% average roughness from purely sandblasted (SB) aluminum surface (Figure 20a). On increasing the AC concentration from 5 wt.% (Ra: 2.7 ± 0.3 µm) to 15 wt.% (Ra: 1.2 ± 0.3 µm), the presence of cavities was visually reduced (Figure 20c). Furthermore, at the highest level of AC concentration considered in this work (25 wt.%), the average roughness decreased by 58%, changing from 1.2 ± 0.3 µm to 0.7 ± 0.2 µm. At this level, the surface was smoother (Figure 20d); this probably helped transfer the stress from the 3D-printed part to the aluminum part, decreasing the local stress concentration, where it led to changes in the failure mode to net-tension. Such a failure mode was along the 45° deposition orientation for these specimens. For the previous conditions investigated, an adhesive failure mode occurred. Hence, as discussed earlier, higher AC improved the mechanical performance of aluminum 2024-T3/ABS hybrid joints.

#### 3.1.5. Number of Contours

**Figure 21:**
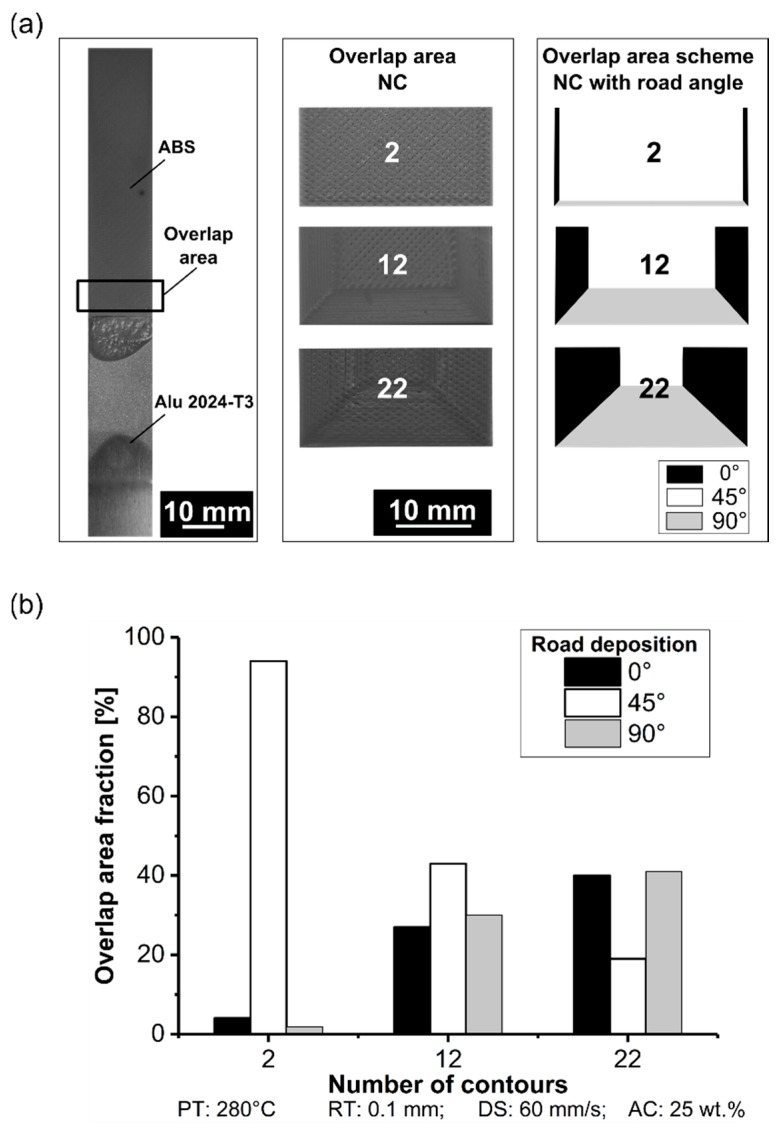
Correlation with the overlap area and road deposition angle on the aluminum 2024-T3/ABS hybrid joints (**a**) schematic, (**b**) overlap area fraction (condition nine, NC: 2; condition ten, NC: 12; condition eleven, NC: 22; the following parameters PT: 280 °C, RT: 0.1 mm, DS: 60 mm/s, and AC: 25 wt.% were fixed for the conditions investigated).

After fixing PT at 280 °C, RT at 0.1 mm, DS at 60 mm/s, and AC at 25 wt.%, NC was evaluated in this section. Increasing NC from its lowest level (condition nine, NC: 2) to the highest level (condition eleven, NC: 22) changed the road distribution arrangement and, consequently, the mechanical performance. Primarily, the infill road orientation was fixed at +45°/−45°; however, with increases in NC, the contribution of the overall road area in 45° was reduced, and the orientation at 0° and 90° increased. The latter two orientations were responsible for the closed loop in the infill pattern, as explained in the previous section (Figure 3 and Figure 17). In the single-lap joint, the force was applied in a tensile direction, which was collinear with the road here referred to as 0°. The overlap area fraction was extracted in the overlap area on the surface of the ABS 3D-printed. Figure 21a shows a schematic view highlighting each of the contributions from 0°, 45°, and 90° road depositions. As the infill road was orientated at +45°/−45°, it contributed positively to the mechanical performance in a similar matter as a 0°-orientated road, a well-known behavior found in traditional composite laminates. The contribution at 0° and 45° are considered together because this orientation represents the positive influence on the mechanical performance. Road aligned with the axis of the load application can help increase the greatest mechanical performance [37,38]. Figure 21b shows that the overlap area fraction for two contours (condition nine) with 0° and 45° reached 4% and 94%, respectively, and 2% for 90°. By increasing to 12 contours (condition ten), the overlapping area fraction increased the contribution of the road deposition in 0° and 90° by 27% and 30%, respectively. At the highest NC (condition eleven), the overlap area fraction was distributed into 40% and 19% for 0° and 45°, respectively, and 41% to 90°.

**Figure 22:**
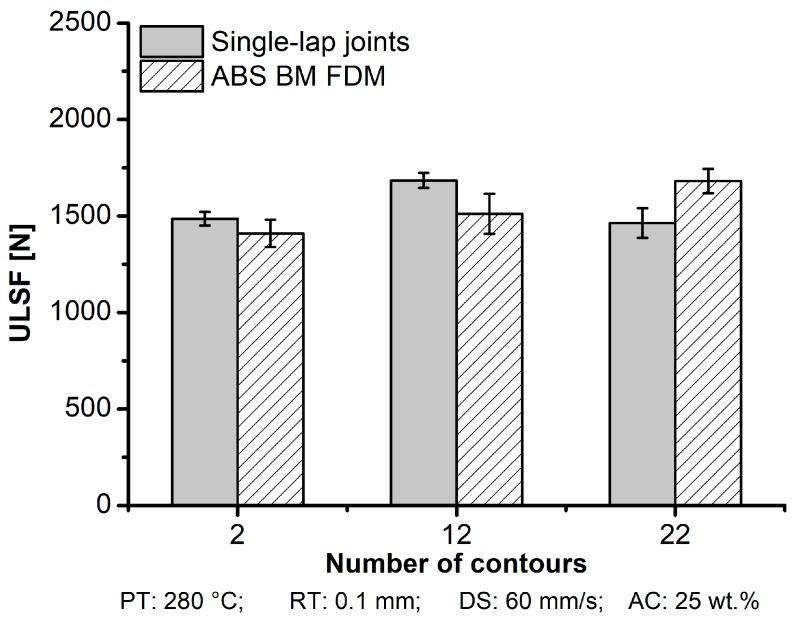
Influence of the number of contours on ULSF for the aluminum 2024-T3/ABS hybrid joints (single-lap joints) and ABS BM FDM (condition nine, NC: 2; condition ten, NC: 12; condition eleven, NC: 22; the following parameters PT: 280 °C, RT: 0.1 mm, DS: 60 mm/s, and AC: 25 wt.% were fixed for the conditions investigated).

The effect of the NC parameter influenced the mechanical performance of aluminum 2024-T3/ABS hybrid joints, and consequently also changed the failure mode. Figure 22 shows that increasing the NC increased the mechanical performance of the single-lap joint. Condition ten (NC: 12) reached the maximum performance (ULSF: 1686 ± 39 N) across the other conditions mentioned. However, by increasing NC further to 22 (Condition 11), a slight decrease in the mechanical performance was observed for the single-lap joint. The explanation lies in the road orientation distribution, where the road deposition at 90° was inevitably formed in the overlap area, and therefore perpendicular to the testing loading direction, which is known to be detrimental.

For the ABS BM FDM, it showed a clear trend of increasing by 17% in the mechanical performance when increasing the number of contours from two (ULSF: 1410 ± 71) to 22 (ULSF: 1682 ± 63). In this work, the average DaB for single-lap joints and a tensile specimen within their range did not statistically change; 2 mm for aluminum 2024-T3/ABS hybrid joints (Table 5) and ABS BM FDM (Table 4) deformed 95% more (3.9 mm). For ABS BM FDM, the road deposition in 90° was inside the grips during mechanical testing, which was far from the carrying loading area. Hence, the influence of road deposition in 90° did not directly influence the performance in the ABS BM FDM.

**Figure 23:**
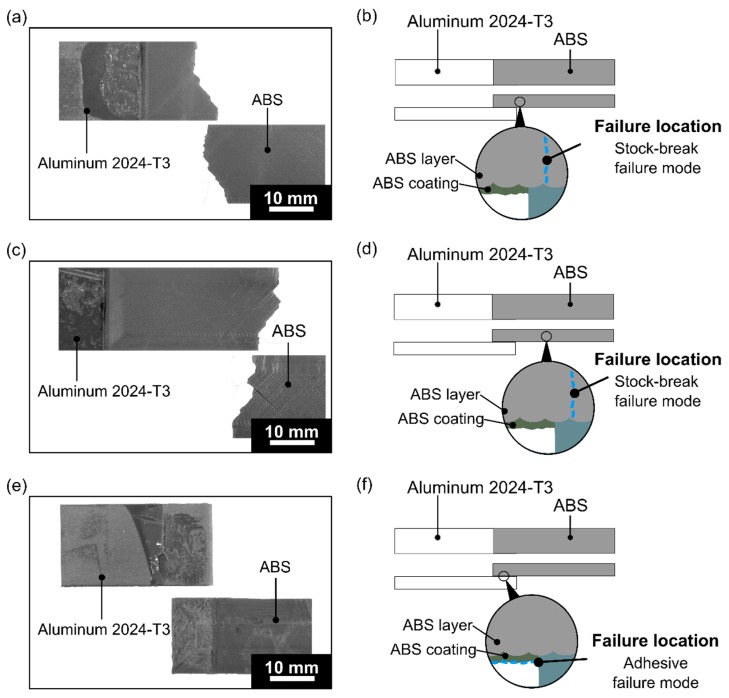
Aluminum 2024-T3/ABS hybrid joint failed joint under a quasi-static lap shear test with its failure mode: (**a**,**b**) NC: 2, (**c**,**d**) NC: 12, and (**e**,**f**) NC: 22 (respectively condition nine, condition ten, and condition eleven; the following parameters PT: 280 °C, RT: 0.1 mm, DS: 60 mm/s, and AC: 25 wt.% were fixed for the conditions investigated).

For the aluminum 2024-T3/ABS hybrid joints, this isolated parameter was the only one with a different failure mode. The previous conditions failed in the interface with a typical adhesive failure (condition one to condition eight from Table 5). Figure 23 shows the different failure mode results influenced by changing NC. For the two contours (Figure 23a,b) and 12 contours (Figure 23c,d), the mode changed to a net-tension failure, where the specimen failure along the 45° degrees was along the road bonding line. The similar pattern at 45° also occurred for the ABS BM FDM. Increasing the number of contours to 22 contributed to a wider contour width area (as shown in Figure 21), which increased the contribution at the 0° orientation by 40%, and primarily the main part to carry the load than by infilling roads. Nevertheless, it also reduced the effectiveness of the load transfer, because it reached 41% of the overlap area with 90° orientation. Hence, for this condition (NC: 22), the contribution for the 90° orientation was weakening the mechanical performance of the single-lap joint. Moreover, the failure mode reached the adhesive failure for the highest level (NC: 22) (Figure 23e,f).

### 3.2. Optimum Condition Based on Maximum ULSF

**Figure 24:**
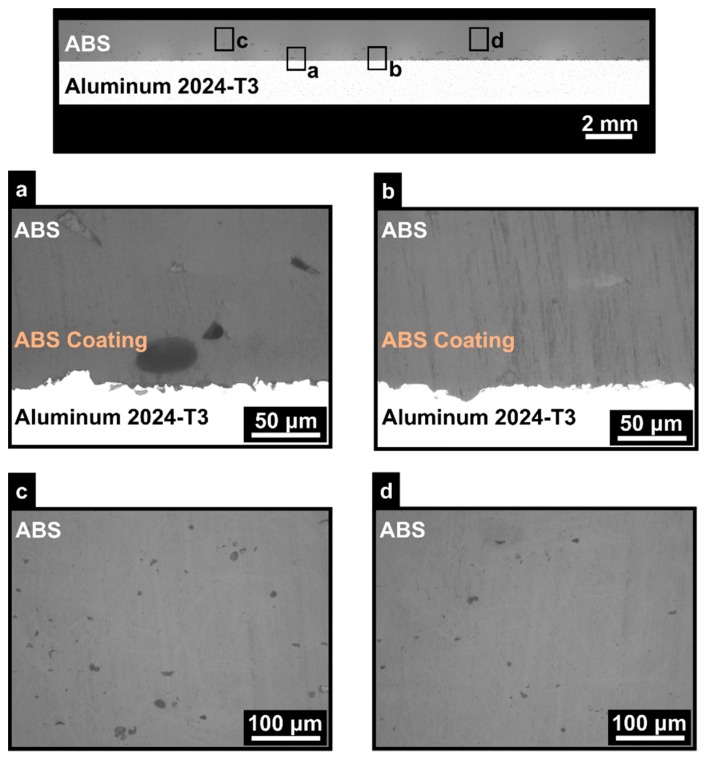
Microstructure for aluminum 2024-T3/ABS hybrid joints obtained by the AddJoining process: (**a**) voids in the ABS coating layer, (**b**) the interface between ABS and the aluminum, and (**c**,**d**) void formation in the printed ABS (PT: 280 °C, RT: 0.1 mm; DS: 60 mm/s; AC: 25 wt.%; NC: 12).

In this section, a condition was selected based on the maximum ULSF for the aluminum 2024-T3/ABS hybrid joints (ULSF: 1686 ± 39 N) from the OFAT (condition ten; PT: 280 °C; RT: 0.1 mm; DS: 60 mm/s; AC: 25 wt.%; NC: 12). Consequently, the joint was analyzed regarding the microstructural features and fracture analysis. The cross-section of the aluminum 2024-T3/ABS hybrid joints is shown in Figure 24. Different features are present in the cross-sectional image, where a detailed analysis was performed using optical microscopy. Voids in the ABS coating can be observed in Figure 24a, with the average cross-sectional area of 2583 ± 1124 µm^2^, due to evaporation of the remaining acetone from the solution during the AddJoining process. Good visual adhesion between deposited ABS and the coated aluminum substrate was achieved (Figure 24b), resulting in strong bond formation at the interface. Moreover, no bond line could be visually detected between deposited ABS and ABS-coated aluminum (ABS coating layer of 98 ± 9 µm). This is an indication of improved intermolecular-diffusion supported by the high deposition temperature. In other words, strong bond formation between the two ABS layers was formed.

**Figure 25:**
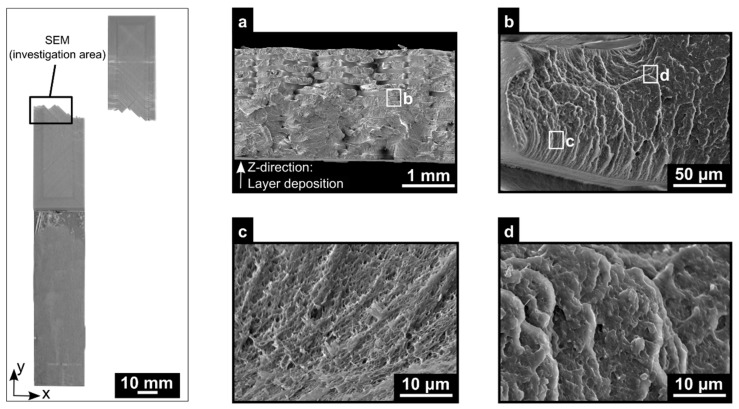
Aluminum 2024-T3/ABS hybrid joint failed joint under a quasi-static lap shear test and SEM images of the fracture surface (**a**) at low magnification in the cross-section with the direction of deposition along the polymer thickness, (**b**) high magnification of the road fractured, (**c**) fibrils in pulling direction, and (**d**) brittle failure (PT: 280 °C, RT: 0.1 mm; DS: 60 mm/s; AC: 25 wt.%; NC: 12).

A net-tension failure mode in the ABS 3D-printed was obtained for the optimum parameter combination (Figure 25). The total printing time to produce the sample was 40 min with the optimum combinations of parameters (120 s per layer were necessary to manufacture a total of 20 layers). By observing the fracture surface, Figure 25a shows that the ABS layers were melted together for the first 16 layers with a printing time of 32 min. As the bottom layers maintained a relatively high temperature for a longer time, deposition of the new layers continued, leading to the intermolecular diffusion between the layers. This promoted ABS diffusion, where it was in agreement with the literature, where the ABS diffusion time was in the range of 390 to 870 s to allow a good intermolecular diffusion [18,34]. In contrast, the last three layers of the ABS 3D-printed were visibly distinct. These layers did not experience the maintenance at higher temperature to reach the diffusion time because the printing time reached the final part. The fracture patterns at the macroscopic level failed along the 45° bonding line [37,39]. Furthermore, the fracture surface was investigated in the microscopic level with an SEM. The fracture surface was predominantly brittle, where on each road, the crack path was driven on each road face (Figure 25b). During the loading, stress resulted in microshearing and high plastic deformation. The presence of fibrils in the pulling direction was observed, where it indicated that crazes were formed, and it developed prior to the ABS that yielded locally (Figure 25c). Moreover, the fracture surface also displayed evidence of brittle failure, with microshearing on the roads (Figure 25d).

## 4. Conclusions

The AddJoining feasibility was demonstrated for the material combination composed of aluminum 2024-T3 and ABS to form hybrid joints. The base material ABS, represented as ABS BM FDM, was investigated to correlate the mechanical performance with the hybrid joints.

The mechanical performance of aluminum 2024-T3/ABS hybrid joints varied from 910 ± 59 N to 1686 ± 39 N. Moreover, for ABS BM FDM, it ranged from 1142 ± 33 N to 1682 ± 63 N. This study showed that four factors were predominantly important for AddJoining hybrid joints under quasi-static loading in the following order of significance: deposition speed, ABS coating concentration, number of contours, and road thickness. For ABS BM FDM, the factors order was in a slightly different order of significance following the number of contours, deposition speed, and road thickness. Based on ANOVA, printing temperature showed no statistically significant influence on the mechanical performance considering the selected confidence level of 95% and in the selected range of variation. Based on the statistical significance in the investigated range for AddJoining hybrid joints, it can be concluded that:

1. At a higher deposition speed (60 mm/s), the road lost the heat to the consolidated neighboring road or the road below. Hence, it facilitated intermolecular diffusion where the deposited road remained softened upon the deposition of the following road. Therefore, it allowed for a better bonding between the layers and promotes a reduction in pores.
2. Higher ABS coating concentration (25 wt.%) increased the coating thickness to nearly 100 µm. Therefore, it had a smoother surface reducing local stress concentration. Moreover, it had more mass to deform, promoting a better interaction of the metal–polymer leading to plastic deformation and low shear and peel stress.
3. For the highest number of contours (22), the carrying load was decreased by the road orientation in the overlap area. The road deposition for 90° was inevitably formed in the overlap area, which caused the joint to turn weaker. For ABS BM FDM, the road deposition for 90° was in the grips during mechanical testing, which was far from the carrying loading area, and which did not directly influence the performance of the specimen.
4. Thinner road thickness (0.1 mm) resulted in considerable inter-bonding strength and compact interactions with the roads, where the low pores formation in the internal structure of the 3D-printed polymer was found.

Nevertheless, at the microscopic level, increasing printing temperature in the considered range in this work led to the promotion of a thermal fusion between the ABS layers and attenuation of the void content in the 3D-printed part.

The ULSF that was obtained with the optimized joining parameters was 1686 ± 39 N from the OFAT (condition ten; printing temperature: 280 °C; road thickness: 0.1 mm; deposition speed: 60 mm/s; ABS coating concentration: 25 wt.%; number of contours: 12), failing by net-tension failure mode with failure pattern along the 45° bonding line. This could be considered a positive output as the final failure took place in the printed base material and not at the metal–film or film–printed polymer interfaces. Finally, from the microstructure point of view, good mechanical interlocking was achieved between the coated metal substrate and printer polymer. Overall, the results of this study may be considered as a base for further understanding of joint formation in layered AddJoining hybrid structures, as well as the general joint mechanical behavior of additive manufactured metal–polymer structures.

## Figures and Tables

**Table 1:** Primary physical and mechanical properties of aluminum 2024-T3 at room temperature [17].

Coeff. of Thermal Expansion (µm/m·°C)	Thermal Conductivity (W/m·K)	Melting Temperature (°C)	Elastic Modulus (GPa)	Tensile Strength (MPa)
24.7	121	500–638	72	480

**Table 2:** Primary physical and mechanical properties of ABS at room temperature [18,19,20].

Coeff. of Thermal Expansion (µm/m·°C)	Thermal Conductivity (W/m·K)	Glass Transition Temperature (°C)	Elastic Modulus (GPa)	Tensile Strength (MPa)
10.1	0.21	94	2.4	26

**Table 3:** AddJoining process parameters range for the OFAT.

Factor	Abbreviation	Unit	Level 1	Level 2	Level 3
Printing temperature	PT	°C	230	255	280
Road thickness	RT	mm	0.1	0.2	0.3
Deposition speed	DS	mm/s	20	40	60
ABS coating concentration	AC	wt.%	5	15	25
Number of contours	NC	-	2	12	22

**Table 4:** Summary of the ULSF and DaB for all 11 conditions for ABS BM FDM.

Condition	PT (°C)	RT (mm)	DS (mm/s)	AC (wt.%)	NC (-)	ULSF (N)	DaB (mm)
C1	230	0.2	40	15	2	1228 ± 132	4.7 ± 0.7
C2	255	0.2	40	15	2	1209 ± 95	5.1 ± 0.3
C3	280	0.2	40	15	2	1259 ± 128	5.7 ± 0.5
C4	280	0.1	40	15	2	1401 ± 30	4.8 ± 0.3
C5	280	0.3	40	15	2	1152 ± 75	4.2 ± 0.2
C6	280	0.1	20	15	2	1142 ± 33	4.5 ± 0.6
C7	280	0.1	60	15	2	1410 ± 71	6.2 ± 0.9
C8	280	0.1	60	5	2	1410 ± 71	6.2 ± 0.9
C9	280	0.1	60	25	2	1410 ± 71	6.2 ± 0.9
C10	280	0.1	60	25	12	1412 ± 104	3.8 ± 0.1
C11	280	0.1	60	25	22	1682 ± 63	4.7 ± 0.7

**Table 5:** Summary of the ULSF and DaB for the 11 conditions in the aluminum 2024-T3/ABS hybrid joints.

Condition	PT (°C)	RT (mm)	DS (mm/s)	AC (wt.%)	NC (-)	ULSF (N)	DaB (mm)
C1	230	0.2	40	15	2	1058 ± 89	1.2 ± 0.3
C2	255	0.2	40	15	2	1121 ± 94	1.3 ± 0.2
C3	280	0.2	40	15	2	1159 ± 50	1.3 ± 0.2
C4	280	0.1	40	15	2	1267 ± 27	1.5 ± 0.5
C5	280	0.3	40	15	2	1062 ± 39	1.3 ± 0.3
C6	280	0.1	20	15	2	910 ± 59	0.9 ± 0.1
C7	280	0.1	60	15	2	1340 ± 47	1.8 ± 0.3
C8	280	0.1	60	5	2	1142 ± 35	0.8 ± 0.4
C9	280	0.1	60	25	2	1486 ± 36	1.9 ± 0.2
C10	280	0.1	60	25	12	1686 ± 39	2.3 ± 0.4
C11	280	0.1	60	25	22	1464 ± 77	2.0 ± 0.7

**Table 6:** ANOVA results for ULSF for aluminum 2024-T3/ABS hybrid joints.

Factor	Unit	Level 1	Level 2	Level 3	*f*-Value	*p*-Value
PT	°C	230	255	280	2.15	1.34 × 10^−1^
RT	mm	0.1	0.2	0.3	5.73	8.0 × 10^−3^
DS	mm/s	20	40	60	32.38	1.24 × 10^−6^
AC	wt.%	5	15	25	29.42	2.43 × 10^−5^
NC	-	2	12	22	15.64	1.46 × 10^−5^

**Table 7:** ANOVA results for ULSF for ABS BM FDM.

Factor	Unit	Level 1	Level 2	Level 3	*f*-Value	*p*-Value
PT	°C	230	255	280	2.15	1.34 × 10^−1^
RT	mm	0.1	0.2	0.3	5.73	8.0 × 10^−3^
DS	mm/s	20	40	60	32.38	1.24 × 10^−6^
AC	wt.%	5	15	25	29.42	2.43 × 10^−5^
NC	-	2	12	22	15.64	1.46 × 10^−5^
