# Correction: Falck, R. et al. Microstructure and Mechanical Performance of Additively Manufactured Aluminum 2024-T3/Acrylonitrile Butadiene Styrene Hybrid Joints by AddJoining Technique. *Materials* 2019, *12*, 864

**DOI:** 10.3390/ma13061460

**Published:** 2020-03-23

**Authors:** Rielson Falck, Jorge F. dos Santos, Sergio T. Amancio-Filho

**Affiliations:** 1 Helmholtz-Zentrum Geesthacht, Center for Materials and Coastal Research, Institute of Materials Research, Materials Mechanics, Solid State Joining Processes, 21502 Geesthacht, Germany; rielson.falck@hzg.de (R.F.); jorge.dos.santos@hzg.de (J.F.d.S.); 2 Institute of Materials Science, Joining and Forming, BMVIT Endowed Professorship for Aviation, Graz University of Technology—TU Graz, Kopernikusgasse 24/1, 8010 Graz, Austria

The authors wish to make the following correction to the paper [1]. Due to identical data in Table 6 and Table 7, replace:

**Table 7:** ANOVA results for ULSF for ABS BM FDM.

Factor	Unit	Level 1	Level 2	Level 3	F-Value	*p*-Value
PT	°C	230	255	280	2.15	1.34 × 10^−1^
RT	mm	0.1	0.2	0.3	5.73	8.0 × 10^−3^
*DS*	mm/s	20	40	60	32.38	1.24 × 10^−6^
*AC*	wt.%	5	15	25	29.42	2.43 × 10^−5^
*NC*	-	2	12	22	15.64	1.46 × 10^−5^

with

**Table 7:** ANOVA results for ULSF for ABS BM FDM.

Factor	Unit	Level 1	Level 2	Level 3	F-Value	*p*-Value
PT	°C	230	255	280	1.28	2.97 × 10^−1^
RT	mm	0.1	0.2	0.3	5.65	1.0 × 10^−2^
DS	mm/s	20	40	60	14.09	1.78 × 10^−6^
NC	-	2	12	22	17.19	2.32 × 10^−5^

These changes have no material impact on the discussion and conclusions of the paper. The authors would like to apologize for any inconvenience caused to the readers by these changes. 

## References

[1] Falck R., dos Santos J.F., Amancio-Filho S.T. (2019). Microstructure and Mechanical Performance of Additively Manufactured Aluminum 2024-T3/Acrylonitrile Butadiene Styrene Hybrid Joints Using an AddJoining Technique. Materials.

